# Determinants of vitamin B12 deficiency in patients with type-2 diabetes mellitus — A primary-care retrospective cohort study

**DOI:** 10.1186/s12875-023-02057-x

**Published:** 2023-04-20

**Authors:** Andrew Kien Han Wee, Rehena Sultana

**Affiliations:** 1 SingHealth Polyclinics, Marine Parade Polyclinic, Blk 80 Marine Parade Central, #01-792, Singapore, 440080 Republic of Singapore; 2 SingHealth Duke-NUS Medical School. Family Medicine Academic Clinical Programme (“FM ACP”), Office of Academic & Clinical Development, 8 College Road, Singapore, 169857 Republic of Singapore; 3 Duke-NUS Medical School, Centre for Quantitative Medicine, 8 College Road, Singapore, 169857 Republic of Singapore

**Keywords:** Metformin, Folate deficiency, Elderly, Vegetarianism, COVID-19, Vitamin B12 deficiency

## Abstract

**Background:**

Like many developed nations, the prevalence of both older people and type-2 diabetes mellitus (T2DM) in Singapore is rising. This demographic shift predisposes the population to greater risks of both frailty and its complications that can be further aggravated by vitamin B12 deficiency —a highly prevalent associated variable that is potentially modifiable.

Indeed, B12 deficiency adversely impacts the neuro-cognitive, haematological, and even the immune systems; jeopardizing our aspirations for successful aging. Despite this, many patients with T2DM in primary care remain unscreened due to a lack of clear guidelines for regular B12 screening. We therefore investigated the determinants of B12 deficiency in community-dwelling patients with T2DM, with the aim of profiling patients most in need of B12-deficiency screening.

**Methods:**

B12 deficiency was evaluated using a retrospective cross-sectional cohort of 592 primary-care patients with T2DM, recruited from 2008 to 2011 from a Polyclinic in Singapore.

**Results:**

B12 deficiency (serum B12 < 150 pmol/L) was present in 164 (27.7%) patients and was associated with a higher “metformin daily dose” (OR = 2.79; 95% CI, 2.22–3.48, *P* < 0.001); “age ≥ 80 years” (OR = 2.86; 95% CI, 1.31–6.25, *P* = 0.008); “vegetarianism” (OR = 21.61; 95% CI, 4.47–104.44, *P* < 0.001); and “folate deficiency” (OR = 2.04; 95% CI, 1.27–3.28, *P* = 0.003). Conversely, “Prescribed B12 supplementation” was associated with a lower odds of B12 deficiency (OR = 0.37; 95% CI: 0.22–0.61, *P* < 0.001). The area under the receiver operating characteristic curve was 0.803 (95% CI: 0.765–0.842).

“Metformin daily dose” correlated interchangeably with “Metformin 1-year cumulative dose” (*r* = 0.960; *P* < 0.01), and also associated linearly with “duration of diabetes” (B = 0.113, *P* < 0.0001).

Independent of the duration of T2DM, 29.3% of the B12-deficient patients needed > 1 screening test before the detection of B12 deficiency.

**Conclusions:**

Primary-care screening for B12 deficiency should be part of the annual laboratory review of patients with T2DM regardless of the duration of T2DM —especially when they are prescribed ≥ 1.5 g/day of metformin; ≥ 80 years old; vegetarian; and not prescribed B12 supplementation. Concurrent evaluation for associated folate (vitamin B9) deficiency is essential when addressing T2DM-associated B12 deficiencies.

Current “Metformin daily dose” is an accurate proxy of both cumulative metformin exposure and duration of T2DM.

**Supplementary Information:**

The online version contains supplementary material available at 10.1186/s12875-023-02057-x.

## Background

Like other developed nations, the prevalence of old age [1] and type-2 diabetes mellitus [2] (T2DM) in Singapore is set to rise appreciably. This demographic trend, in turn, predisposes the population to greater risks of frailty [3] and its complications, that could be further compounded by vitamin B12 deficiency [4]—a well-known associated variable that is prevalent among the elderly and those with T2DM, but yet potentially modifiable.

Vitamin B12 deficiency (hereinafter referred to as “B12” deficiency) has wide-reaching consequences; its deleterious impact on the neuro-cognitive, haematological and even cardiovascular systems is well-described [5–8]. There is also emerging evidence that vitamin B12 could modulate the immune system, possibly affecting how we respond to COVID-19 infections [4, 9], or even to vaccination [10]. Our long-term aspirations for both successful aging and resilience to emerging infectious diseases could thus be jeopardized by vitamin B12 deficiency.

Many of the published reasons for B12 deficiency’s association with T2DM relate to everyday clinical care. Metformin —the mainstay of type-2-diabetes treatment [11, 12] —is strongly associated with B12 deficiency [13, 14]. Metformin reportedly disrupts calcium-dependent intestinal absorption of vitamin B12 (that is reversed by calcium supplementation) [15]. Elderly patients with T2DM could also risk having B12 deficiency because of poorer nutrition; diets weighted on vegetables [5–7]; or B12 malabsorption [16]. The regular use of either proton-pump inhibitors (PPIs) or histamine H2 receptor antagonists (H_2_As) among these patients [5, 6] —especially as an adjunct to aspirin therapy in the secondary prevention of cardiovascular events [17, 18] —could also reduce the ability to digest B12 from food [19].

The central role of primary care (vis-à-vis specialist care) in the provision of quality and preventive health in the community is increasingly being acknowledged [20, 21]. Primary-care studies of patients with T2DM have consistently shown a prevalence of B12 deficiency in excess of 20% [22, 23]. Despite this, screening for T2DM-associated B12 deficiency in clinical practice is still lacking; an Irish audit of 241 patient records in 2019 (within both primary and specialist care) found that 56 (23%) patients had never been screened for B12 levels [24]. Moreover, there is still no clear guideline on regular screening for B12 deficiencies in primary care [25]. Instead, current guidelines on T2DM remain vague and only recommend “periodic” [12] testing for B12 deficiencies in the limited context of patients taking “metformin” [11, 12, 26], and only when they have “established anaemia or peripheral neuropathy” [11, 26] —serious complications that are often late or even irreversible; complications that ought to have been prevented in the first place.

Although there is considerable literature on the individual determinants of B12 deficiency in T2DM, how these determinants combine to associate with B12 deficiency in a primary-care-T2DM setting is still unclear. A better understanding could help us profile and identify the patients most in need of B12-deficiency screening in the community; ultimately enabling us to prevent complications of deficiency early and cost-effectively.

We therefore conducted a retrospective cohort [27] study primarily to find out the variables associated with B12 deficiency within a multiethnic primary care setting of Singapore. We also sought to explore and narrow down which aspect of metformin (dose; duration; or a combined cumulative exposure) had the greatest relation to B12 deficiency [25]; knowledge of which could simplify the screening process in a busy primary-care setting.

## Methods

### Study population and covariates

Primary care in Singapore is delivered through 1800 private general-practice clinics and 23 public polyclinics [28]. In 2014, the polyclinics handled 41% of chronic attendances to primary care, of which 32% were for the principal diagnosis of diabetes mellitus [29].

A list of all the patients with T2DM who were registered to the Family Physician Clinic (FPC) service from January 2008 to July 2011 was first made. Family physicians with at least a post-graduate qualification in family medicine run this enhanced FPC service. The patients in the FPC differ from those in the clinic’s general pool in that the fees for consultation and medication —though still highly subsidised for Singapore citizens and permanent residents —are higher. Patients in the FPC are able to choose and see the same family physician on an appointment basis, enhancing the continuity of care [30, 31]. On the other hand, the patients in the general pool are assigned to whichever physician, and of varying seniority, available during their visit (this may include FPC physicians who were are not rostered to run the FPC for that visit).

The FPC service in our clinic had piloted periodic [12] testing of B12-folate levels in patients with T2DM since 2008 [32], but no screening protocol was used with regard to either the frequency of testing, when to test, or on whom to test that is in keeping with the current guideline [12]. This pragmatic approach meant that all the patients with T2DM had to have at least one B12-level screening test result on record, regardless of their perceived risk for deficiency (eg age, gender, ethnicity, dietary restrictions, duration of diabetes, concurrent medication, use of supplements, all aspects of metformin use, etc.). When B12 levels were in the low range of normality (i.e. between 150 to 200 pmol/L), tests for B12 levels were ordered more frequently.

A total of 608 patients’ records were obtained, of which 592 were included into the study: 14 patients were excluded because they had no prior test for serum B12-folate level; 2 were excluded because one was diagnosed to have type-1 diabetes mellitus and the other had a missing LDL cholesterol level test result. If there were multiple tests for B12 levels, the lowest B12 level was chosen and the date of this result was designated the “reference B12 test date”. No other exclusion criterion was used — to better maximize the external validity of our findings.

For all the subjects, information for covariates were extracted from the electronic clinical records for the 12-month period leading to the reference B12 test dates. These include measures of metformin exposure (metformin daily dose; metformin 1-year cumulative dose); age; gender; ethnicity; body mass index (BMI); vegetarianism; prescribed supplementation (for B12 and calcium supplements); use of both proton pump inhibitors (PPIs) and/or histamine-2 antagonists (H_2_A); concurrent serum folate level; serum creatinine; serum LDL cholesterol; glycated haemoglobin (HbA1c); and the use of lipid-lowering; blood-pressure-lowering; and diabetic medication.

Other covariates include the duration of T2DM (< 1 year; yearly up to 9 years; and ≥ 10 years); duration of metformin use (none to 6 months; > 6 months to 1 year; > 1 year to 2 years; > 2 years); and the number of times the patient was tested for B12 deficiency (before the reference B12 test).

All patients in the FPC are routinely asked if they are “vegetarian”; and their case records would be updated accordingly to indicate this self-defined dietary restriction. The various types of vegetarianism (eg lacto-ovo; vegan; or semi- [33, 34]) were however not distinguished in the clinical notes. We used the computerised-prescription platform shared by the SingHealth cluster of hospitals, specialist centres, and polyclinics to obtain both prescribed medication and prescribed supplementation details. Herein, any computer record for either prescribed medication (eg PPI) or prescribed supplementation (eg B12) within the 12 months leading up to the reference B12 test date —notwithstanding the mode of delivery or duration of use within that year —was considered positive for use. With the exception of B12-folate level (which were ordered periodically as described above), all other laboratory tests were done at least annually as part of routine diabetic care.

This study was conducted in Marine Parade Polyclinic according to the guidelines set out in the Declaration of Helsinki; SingHealth Centralised Institutional Review Board had granted both ethical approval and full waiver from written informed consent (CIRB Reference: 2011/437/E).

### Measurements

“Metformin daily dose” (g/day) was obtained by taking the most recent prescription that was effective at the reference B12 test date. This variable, although convenient to obtain, is only a cross-sectional derivative of a patient’s metformin exposure. We therefore also explored the cumulative metformin exposure by obtaining the “Metformin 1-year cumulative dose” (g/year). This was done by summing up the cumulative dose of metformin consumed within the preceding year leading to the reference B12 test date.

#### Laboratory measurements

Serum vitamin B12 and corresponding folate levels were analysed by Access Immunoassay Systems 33,000 and A98032, respectively (Beckman Coulter, Brea, CA, USA); LDL cholesterol was measured indirectly using the Friedewald equation; total cholesterol was measured with CHO2I: ACN 8798 and CHO2A: ACN 8433 in vitro tests; triglycerides were measured with the TRIGL: ACN 8781 in vitro test; cobas® analyser; HbA1c was measured with the A1-W3: ACN 881 in vitro test on a cobas® analyser; creatinine was measured with the CREJ2: ACN 8690 in vitro test; cobas® analyser while the estimated glomerular filtration rate (eGFR) was derived using the Modification of Diet in Renal Disease Study (MDRD) equation.

B12 deficiency was defined as B12 level < 150 pmol/L [5, 6, 35, 36] whilst folate deficiency was defined as a folate level ≤ 13.4 nmol/L [37].

### Statistical analyses

Outcome B12 deficiency was treated as binary variable with status “*normal”* or “*B12 deficiency*”. The baseline variables were summarized based on B12 deficiency status. Continuous and categorical variables were summarized using mean (± standard deviation (SD)) and frequency (percentage) respectively. Differences between statuses of B12 deficiency were compared using student’s t-tests and Fisher’s exact tests for continuous and categorical data respectively.

Linear correlation between “Metformin daily dose” and “Metformin 1-year cumulative dose” were determined using the Pearson’s correlation coefficient.

Univariate and subsequent multivariable logistic regression models were then performed to find associated factors for outcome B12 deficiency. Associations from logistic regression were expressed in terms of odds ratio (OR) with 95% confidence interval (95% CI). Variables with *P* values < 0.1 in univariate analyses and four clinically important variables (age, PPI or H_2_A use, gender [38] `and ethnicity [39]) were selected for multivariable model. Stepwise, forward and backward variable selection methods were used to finalize the final multivariable model.

Age was categorised into 3 groups of “ < 60 years”, “60–79 years” and “ ≥ 80 years” to reflect the young; young-old/middle-old; and very old age groups respectively [40].

For daily metformin dose, the “nearest” method [41] was used in conjunction with receiver operator characteristic (ROC) curve analyses for B12 deficiency to analyse its cut-point value (which demarcates the optimal concurrent sensitivity and specificity for B12 deficiency).

All tests were two-sided and P value < 0.05 was considered as statistical significance. The STATA 13.0 statistical software (Stata Corporation, College Station, TX) was used to perform the analyses.

We had 592 analyzable patients. Our primary objective was to find associated risk factors for “B12 deficiency” group. Peduzzi et al., Concato et al. and Vittinghoff et al. recommended that multivariable logistic regression models should be used with at least 10 events per predictor variable [42–44]. We had 15 clinically meaningful variables to account for in the multivariable model and hence we needed at least 10*15 = 150 events in the data. In our data, prevalence of “B12 deficiency” group were at least 27% i.e. we had more than 150 patients in “B12 deficiency” group. Our study was adequately powered (> 80%) with 592 patients based on following assumptions: proportion of “B12 deficiency” as 25%, OR of 1.8 (or 0.56) and alpha or type I error rate as 5%.

## Results

### Baseline characteristics

Table 1 summarizes the baseline characteristics of patients by B12 status; herein, 27.7% (164 of the 592 patients) had B12 deficiency while 22.3% (132 patients) were folate-deficient. Folate deficiency was more prevalent in B12-deficienct patients; it was present in 31.7% (52 of 164) of B12-deficient patients (representing patients deficient in both B12 and folate), compared to only 18.7% (80 of 428) of B12-replete patients (*P* = 0.001).

**Table 1:** Patient characteristics of patients according to vitamin B12 status (*n* = 592)

**Characteristics**	Total (*n* = 592)	Vitamin B12 levels		*P – value*^a^
		Normal (≥ 150 pmol/L) (*n* = 428)	Deficiency (< 150 pmol/L) (*n* = 164)	
Age				
Age (years), mean (± SD)	66.52 (± 10.82)	66.72 (± 10.59)	66.01 (± 11.42)	0.476
Age groups, n (%)				
< 60 years	137 (23.14)	92 (21.50)	45 (27.44)	0.249
60–79 years	390 (65.88)	290 (67.76)	100 (60.98)	
≥ 80 years	65 (10.98)	46 (10.75)	19 (11.59)	
Gender, *n* (%)				
Men	267 (45.10)	188 (43.93)	79 (48.17)	0.358
Women	325 (54.90)	240 (56.07)	85 (51.83)	
Ethnicity, *n* (%)				
Chinese	454 (76.69)	332 (77.57)	122 (74.39)	0.232
Malay	42 (7.09)	32 (7.48)	10 (6.10)	
Indian	72 (12.16)	45 (10.51)	27 (16.46)	
Eurasian & others	24 (4.05)	19 (4.44)	5 (3.05)	
Physical parameters, mean (± SD)				
Height (m)	1.595 (± 0.096)	1.595 (± 0.093)	1.595 (± 0.104)	0.948
Weight (kg)	65.69 (± 14.64)	65.36 (± 14.40)	66.52 (± 15.27)	0.390
BMI (kg/m^2^)	25.72 (± 4.76)	25.60 (± 4.74)	26.03 (± 4.83)	0.326
Chronic Disease, *n* (%)				
Duration of diabetes mellitus ≥ 10 years	296 (50.00)	201 (46.96)	95 (57.93)	0.022
Hypertension	515 (86.99)	374 (87.38)	141 (85.98)	0.683
Hyperlipidemia	540 (91.22)	389 (90.89)	151 (92.07)	0.747
Ischemic heart disease	120 (20.27)	86 (20.09)	34 (20.73)	0.909
Stroke	73 (12.33)	52 (12.15)	21 (12.80)	0.889
Cancer	56 (9.46)	40 (9.35)	16 (9.76)	0.876
Lifestyle, *n* (%)				
Vegetarianism	14 (2.36)	2 (0.47)	12 (7.32)	< 0.001
Vitamin-Mineral Supplementation, *n* (%)				
Prescribed vitamin B12 supplementation	188 (31.76)	157 (36.68)	31 (18.90)	< 0.001
Prescribed calcium supplementation	221 (37.33)	177 (41.36)	44 (26.83)	0.001
Medication, *n* (%)				
Proton-pump inhibitor (PPI) or histamine 2 antagonist (H_2_A) use	124 (20.95)	91 (21.26)	33 (20.12)	0.822
Statin use	499 (84.29)	358 (83.64)	141 (85.98)	0.530
Fenofibrate use	60 (10.14)	39 (9.11)	21 (12.80)	0.223
Metformin use in preceding year	438 (73.99)	293 (68.46)	145 (88.41)	< 0.001
**Metformin 1-year cumulative dose** (g/preceding year), mean (± SD)	436.18 (± 378.75)	350.73 (± 348.12)	659.20 (± 365.28)	< 0.0001
**Metformin daily dose** (g/day), mean (± SD)	1.25 (± 1.04)	1.00 (± 0.96)	1.90 (± 0.99)	< 0.0001
Sulphonylurea use	291 (49.16)	183 (42.76)	108 (65.85)	< 0.001
Dipeptidyl peptidase-4 inhibitors use	29 (4.90)	22 (5.14)	7 (4.27)	0.832
Acarbose (alpha-glucosidase inhibitor) use	85 (14.36)	41 (9.58)	44 (26.83)	< 0.001
Insulin use	57 (9.63)	36 (8.41)	21 (12.80)	0.119
Laboratory Measurements, mean (± SD)				
Vitamin B12 level (pmol/L)	244.13 (± 150.22)	296.30 (± 145.12)	107.98 (± 29.32)	< 0.0001
Folate level, (nmol/L)	24.59 (± 12.92)	25.74 (± 12.84)	21.58 (± 12.71)	< 0.001
Folate deficiency, n (%)	132 (22.30)	80 (18.69)	52 (31.71)	0.001
HbA1c (%)	7.11 (1.08)	7.03 (± 1.03)	7.32 (± 1.17)	< 0.01
Creatinine (µmol/L)	82.14 (± 31.63)	82.11 (± 32.25)	82.23 (± 30.05)	0.966
eGFR using the MDRD study equation (mL/min/1.73m^2^)	77.61 (± 23.59)	77.61 (± 23.69)	78.48 (± 24.48)	0.695
LDL cholesterol level (mmol/L)	2.36 (0.65)	2.40 (± 0.67)	2.27 (± 0.60)	0.034

*Abbreviations*: *BMI* body mass index, *eGFR* estimated glomerular filtration rate adjusted for body surface area, *HbA1c* glycated hemoglobin, *LDL* low-density lipoprotein, *MDRD* Modification of Diet in Renal Disease, *SD* standard deviation

^a^ Fisher’s exact test for the categorical variables and *t*-test for the continuous variables were used

“Metformin daily dose” showed the most significant baseline association with B12 deficiency; B12-replete patients were taking a mean (± SD) of 1.00 (± 0.96) g/day while B12-deficient patients took a mean of 1.90 (± 0.99) g/day, *P* < 0.0001.

**Fig. 1:**
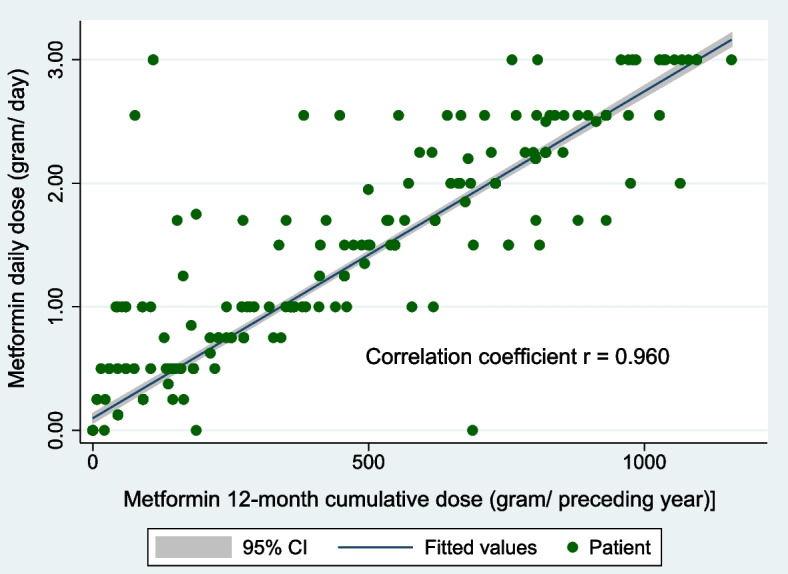
Correlation of “Metformin daily dose” and “Metformin 1-year cumulative dose” (*n* = 592). The 1^st^ variable gives a convenient cross-sectional estimate of metformin exposure whilst the 2^nd^ variable gives a more accurate cumulative measure of metformin exposure

Both “Metformin daily dose” and “Metformin 1-year cumulative dose” were highly correlated (*r* = 0.960, *P* < 0.001) (Fig. 1). Hence, “Metformin 1-year cumulative dose” was excluded from multivariable logistic regression to avoid multicollinearity between the two variables.

### Logistic regression analyses for B12 deficiency (Table 2)

**Table 2:** Logistic regression of B12 deficiency on potential determinants (*n* = 592)

Variables	**Univariate** logistic regression		**Multivariable** logistic regression	
	OR (95% CI)	*P* value	OR (95% CI)	*P* value
Age group^a^ (Reference: < 60 years)		0.259 +		0.027
60–79 years	0.70 (0.46 – 1.08)	0.105	1.26 (0.77 – 2.07)	0.357
≥ 80 years	0.84 (0.44 – 1.61)	0.606	2.86 (1.31 – 6.25)	0.008
Vegetarian^a^ (Reference = non-vegetarian)	16.82 (3.72 – 76.00)	< 0.001	21.61 (4.47 – 104.44)	< 0.001
B12 supplementation (prescribed)^a^	0.40 (0.26 – 0.62)	< 0.001	0.37 (0.22 – 0.61)	< 0.001
Metformin daily dose^a^ (g/day)	2.44 (2.00 – 2.97)	< 0.001	2.79 (2.22 – 3.49)	< 0.001
Folate deficiency^a^	2.02 (1.34 – 3.04)	< 0.001	2.04 (1.27 – 3.28)	0.003
LDL cholesterol level^a^ (mmol/L)	0.73 (0.54 – 0.98)	0.031		
Ethnicity^a^ (Reference: Chinese)		0.220 +		
Malay	0.85 (0.41 – 1.78)	0.668		
Indian	1.63 (0.97 – 2.75)	0.065		
Others	0.72 (0.26 – 1.96)	0.516		
Male gender^a^ (Reference = Female)	1.19 (0.83 – 1.70)	0.353		
Duration of diabetes for ≥ 10 years^a^	1.55 (1.08 – 2.24)	0.017		
Calcium supplementation (prescribed)^a^	0.52 (0.35 – 0.77)	< 0.001		
PPI or H_2_A use^a^	0.93 (0.60 – 1.46)	0.760		
Sulphonylurea use^a^	2.58 (1.77 – 3.76)	< 0.001		
Acarbose use^a^	3.46 (2.16 – 5.55)	< 0.001		
HbA1c^a^ (%)	1.26 (1.07 – 1.48)	< 0.01		
Statin use	1.20 (0.72 – 2.00)	0.481		
Fenofibrate use	1.46 (0.83 – 2.57)	0.192		
Insulin use	1.60 (0.90 – 2.83	0.114		
DPP4-inhibitor use	0.82 (0.34 – 1.97)	0.656		
Creatinine (µmol/L)	1.00 (0.99 – 1.01)	0.966		
eGFR (mL/min/1.73m^2^)	1.00 (0.99 – 1.01)	0.694		
Hypertension	0.89 (0.52 – 1.50)	0.651		
Hyperlipidaemia	1.16 (0.60 – 2.24)	0.645		
Body Mass Index (kg/m^2^)	1.02 (0.98 – 1.06)	0.328		

*Abbreviation*: *eGFR*, estimated glomerular filtration rate adjusted for body surface area

^a^Predictor variables that were further analysed within multivariable logistic regression. + refers to omnibus *p* – value. “Age”, “Ethnicity”, “Gender” and “PPI or H_2_A use” were predetermined to be clinically or demographically relevant, and thus forced into the modelling process

Univariate logistic regression (Table 2) showed that “Metformin daily dose”; “vegetarianism”; “prescribed B12 supplementation”; “folate deficiency”; “LDL cholesterol levels”; having a “history of diabetes for ≥ 10 years”; “prescribed calcium supplementation”; “HbA1c”; “sulphonylurea use”; and “acarbose use” were significantly associated with B12 deficiency.

The univariate cut-point on the ROC curve for “daily metformin dose” was 1.425 g/day; area under the receiver operating characteristic curve (AUROC) = 0.739 (95% CI: 0.694–0.785); (sensitivity 83.86%; specificity 64.95%).

Multivariable logistic regression reduced the significant predictors of B12 deficiency to the following: “Metformin daily dose” (adjusted OR = 2.79; 95% CI, 2.22–3.48, *P* < 0.001); “vegetarianism” (adjusted OR 21.61; 95% CI, 4.47–104.44, *P* < 0.001); “age ≥ 80 years” (adjusted OR = 2.86; 95% CI, 1.31–6.25, *P* = 0.008); “prescribed B12 supplementation” (adjusted OR = 0.37; 95% CI, 0.22–0.61, *P* < 0.001); and “folate deficiency” (adjusted OR = 2.04; 95% CI, 1.27–3.28, *P* = 0.003). The AUROC of the multivariable model was 0.803 (95% CI: 0.765–0.842) and there was no significant interaction between the significant independent variables.

**Fig. 2:**
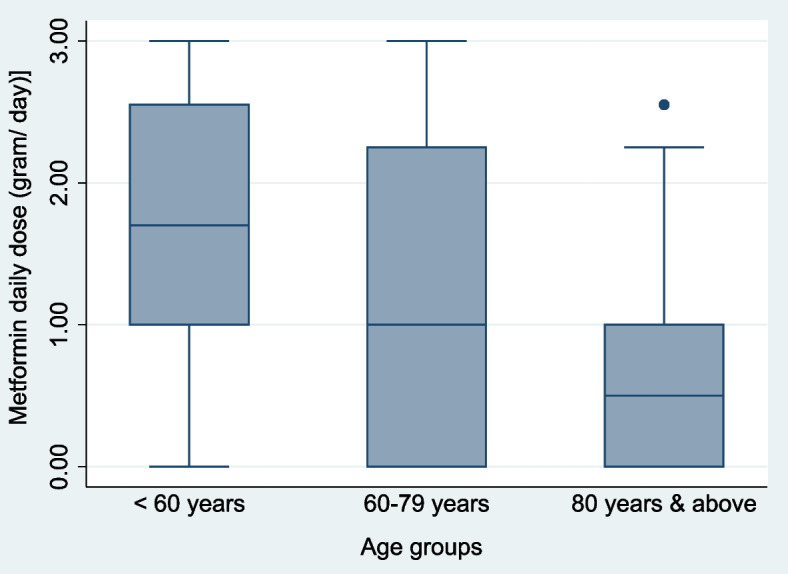
Box plot of Metformin daily dose (g/day) by age groups (*n* = 592). This shows the oldest age group (age ≥ 80 years) taking significantly less metformin daily than the youngest age group (age < 60 years) (*P* < 0.001); Age is thus a confounder in Metformin daily dose’s association with B12 deficiency

Because “age ≥ 80 years” significantly associated with B12 deficiency in multivariable logistic regression (and not within univariate regression), we explored “age ≥ 80 years” successively with each significant predictor of B12 deficiency and found “Metformin daily dose” to be a negative confounding variable. Patients in the older age groups were more likely to take lower daily doses of metformin: with mean (± SD) of 1.69 (± 0.97) g/day; 1.19 (± 1.04) g/day; and 0.69 (± 0.82) g/day of metformin for patients “ < 60 years”; “60–79 years”; and “ ≥ 80 years” respectively (Fig. 2). The differences in the means were statistically significant within one-way ANOVA (F = 24.20, *P* < 0.001).

In addition, because “use of acarbose”, “use of sulphonylureas”, and “HbA1c” predicted B12 deficiency only in univariate analyses (and not in multivariable logistic regression), we further analyzed and found that both the “use of acarbose” and “sulphonylureas” were each associated with higher “metformin daily doses”. Patients on acarbose were taking a mean (± SD) of 2.29 (± 0.871) g/day of metformin compared to 1.08 (± 0.967) g/day of metformin in patients not on acarbose (*P* < 0.0001). Likewise, those taking sulphonylureas were on 1.73 (± 1.01) g/day of daily metformin compared to 0.79 (± 0.858) g/day for those not taking sulphonylureas (*P* < 0.0001).

Similarly, we found that “HbA1c” was also positively associated with metformin: patients taking any dose of metformin had a higher “HbA1c” mean (± SD) of 7.25 (± 1.04) % compared to patients not on metformin: HbA1c of 6.72 (± 1.08) %; (*P* < 0.0001). Regression analysis also showed “HbA1c” associating with “metformin daily dose” in a linear fashion (B = 0.037, *P* < 0.001).

The prevalence of respective CKD stages (by GFR) [45] in our cohort was CKD 1 (29.22%; 173 patients); CKD 2 (50.17%; 297 patients); CKD 3a (11.15%; 66 patients); CKD 3b (7.26%; 43 patients); CKD 4 (2.03%; 12 patients); and CKD 5 (0.17%; 1 patient).

Sensitivity analyses of CKD (as a possible confounder for B12 deficiency) were done by forcing “eGFR” (a continuous variable representing kidney function) into the multivariable logistic regression model, and doing the same with “CKD Stage” (an ordinal variable representing kidney function). Both analyses showed neither of these proxies of kidney function to be significant predictors of B12 deficiency (data not shown). Moreover, when we analysed only patients with CKD stages 1, 2 and 3a (where the eGFR is ≥ 45 mL/min/1.73m^2^; wherein metformin use is not restricted, [46]) the multivariable model we propose was unaffected (in that all the variables remained significantly associated with B12 deficiency) (data not shown).

**Fig. 3:**
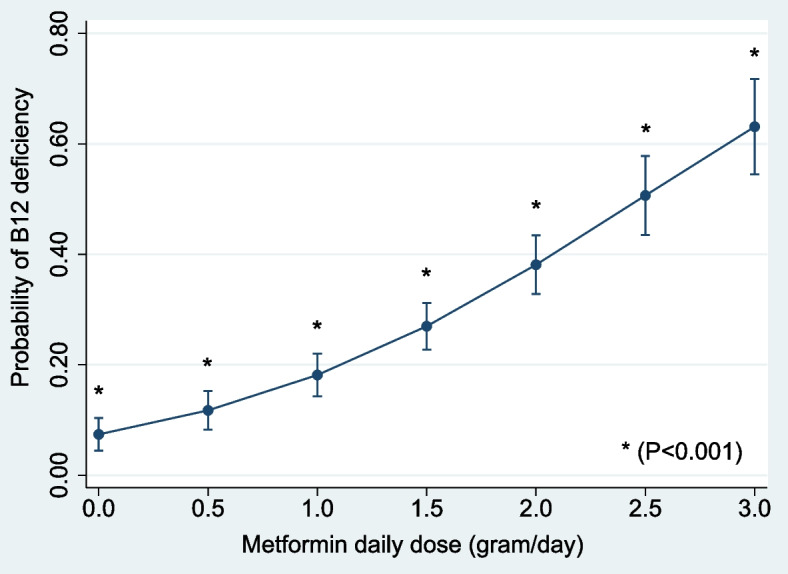
Adjusted probability of “B12 deficiency” by “metformin daily dose” (g/day) (with 95% confidence intervals; *n* = 592). The probabilities are adjusted for age groups, vegetarianism, folate deficiency and prescribed B12 supplementation; showing increasing probability of B12 deficiency at higher daily doses of metformin

**Fig. 4:**
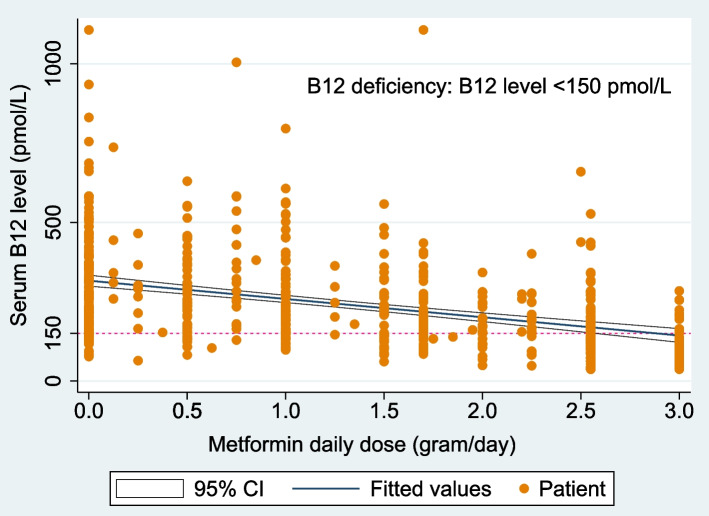
Scatterplot of serum B12 levels and their corresponding daily metformin doses (g/day) (*n* = 592). This shows the mean serum B12 levels (with 95% confidence intervals) of patients decreasing consistently as they take higher daily doses of metformin

“Metformin daily dose” ranged from 0.0 g/day to 3.0 g/day with a mean (± SD) of 1.25 (± 1.04) g/day, and this variable was associated with an almost linear increase in the estimated probability of B12 deficiency at doses above 1.5 g/day (Fig. 3). The estimated probability of B12 deficiency rose with increasing doses of daily metformin: 0.074 (95% CI: 0.044–0.103) at 0.0 g/day of metformin; 0.181 (95% CI: 0.143–0.220) at 1.0 g/day of metformin; 0.270 (95% CI: 0.227–0.312) at 1.5 g/day of metformin; 0.381 (95% CI: 0.328–0.434) at 2.0 g/day of metformin; up to a maximum of 0.631 (95% CI: 0.545–0.717) at 3.0 g/day of metformin. Similarly, Fig. 4 also shows a consistent decline in B12 levels with increasing daily doses of metformin.

**Fig. 5:**
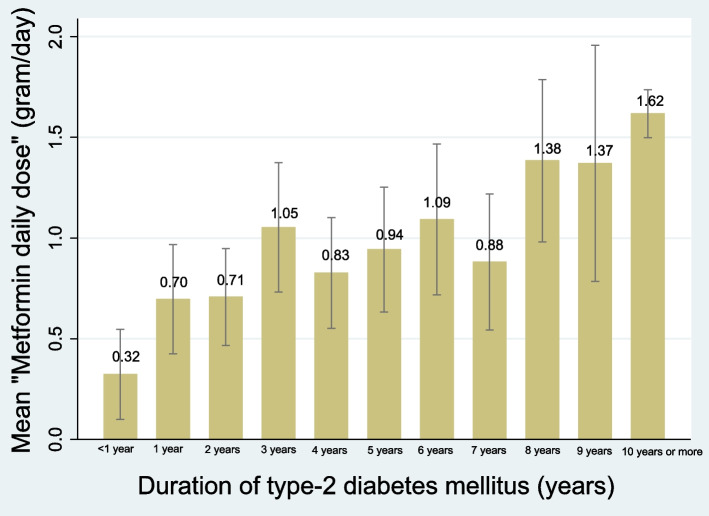
Mean “Metformin daily dose” by the “duration of type-2 diabetes mellitus” (with 95% confidence intervals; *n* = 592). This shows a strong linear association in bivariate regression (B = 0.113, F(1,590) = 99.74, *P* < 0.0001)

“Duration of diabetes (< 10 years vs. ≥ 10 years)” was not a predictor, but rather a confounder of B12 deficiency in multivariable analysis (Table 2). In bivariate regression, we found that “metformin daily dose” strongly associated with “duration of diabetes (< 1 year; yearly up to 9 years; and ≥ 10 years)” (B = 0.113, F(1,590) = 99.74, *P* < 0.0001) (Fig. 5).

**Fig. 6:**
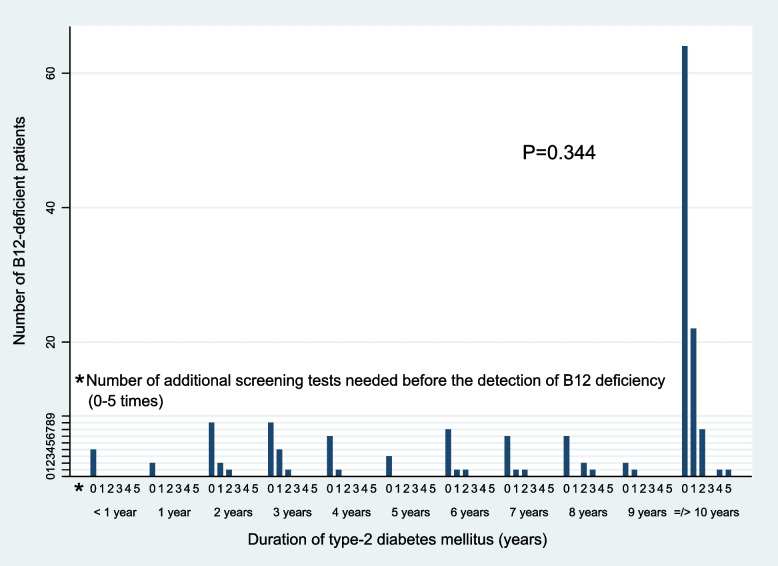
Number of additional screening tests needed before the diagnosis of B12 deficiency (through the “reference B12 test”) by duration of type-2 diabetes mellitus (*n* = 164 B12-deficient patients). This indicates the need for repeated screening of these patients for B12 deficiency

Nevertheless, we sought to explore if “duration of diabetes (< 1 year; yearly up to 9 years; and ≥ 10 years)” had an impact on the frequency of screening tests needed to detect B12 deficiency as defined in this study. Figure 6 shows the distribution of B12-deficient patients by the duration of their T2DM. Of the 164 B12-deficient patients, 48 patients (29.3%) needed one or more preceding B12 screening tests before the detection of B12 deficiency at its lowest level (through the reference B12 test as defined in this study). There was no statistically-significant difference in the number of additional screening tests needed to detect B12 deficiency according to the duration of T2DM (f(10) = 1.13, *P* = 0.344).

## Discussion

Seeking to evaluate the determinants of B12 deficiency in patients with T2DM, we used a retrospective cross-sectional cohort of 592 outpatients with T2DM and found that more than a quarter (27.7%) had B12 deficiency, which was indeed strongly associated with “metformin daily dose” (adjusted OR = 2.79; 95% CI, 2.22–3.49, *P* < 0.001) (Table 2).

Almost 1 in 3 patients who were taking a “metformin daily dose” of 1.5 g/day had B12 deficiency. This rose to 2 in 3 patients who were taking the maximal dose of 3.0 g/day. On the univariate ROC curve for B12 deficiency, “metformin daily dose” had a cut-point of 1.425 (i.e. approximately 1.5) g/day; indicating this to be the threshold value of “metformin daily dose”, beyond which the need for increased clinical vigilance for B12 deficiency is warranted.

Our results are consistent with 2 earlier Asian, albeit ethnically homogenous and non-primary-care-based studies: the 1^st^ from Hong Kong in 2006 [32] based on 155 B12-deficient patients matched with 310 B12-replete controls sourced from a national central laboratory database; and the 2^nd^ from South Korea in 2014 [47] based on 799 patients (76 of whom were B12-deficient) from a hospital-based tertiary-care diabetes centre. The Hong Kong study showed that a 1 g/day increment of metformin dose conferred a comparable OR of 2.88 (95% CI, 2.15–3.87, *P* < 0.001) whilst the Korean study showed that patients taking 1–2 g/day of metformin —compared to those taking < 1 g/day of metformin —had an adjusted OR for B12 deficiency of 2.52 (95% CI, 1.27–4.99, *P* = 0.008). The Korean study also showed —on the ROC curve of B12 deficiency regressed on daily metformin dose —a comparable cut-off value of 1.125 g/day (sensitivity 64%; specificity 65%) for the daily dose of metformin.

Although “Metformin 1-year cumulative dose” directly reflects the cumulative metformin exposure leading to B12 deficiency [25], we found that it correlates highly and interchangeably (*r* = 0.960) with “metformin daily dose” (Fig. 2). In addition, we also found that “metformin daily dose” associates linearly with “duration of diabetes” (B = 0.113, *P* < 0.0001) (Fig. 6). We therefore propose that “metformin daily dose” (which can be calculated immediately in the primary-care setting) is an accurate proxy of both cumulative metformin exposure and duration of T2DM.

Metformin, as the mainstay of T2DM treatment [11, 12], is almost always started first in patients with early stages of T2DM. As their disease progress through the years — and the control of their T2DM (via HbA1c) becomes increasingly difficult— the doses of metformin are gradually escalated in tandem with the introduction of other classes of medication for T2DM, like sulphonylureas and acarbose [11, 12]. Hence, through their strong association with higher metformin daily usage, it is not surprising to see “acarbose use”, “sulphonylurea use” and “HbA1c” being confounding variables for B12 deficiency.

Metformin is excreted renally and its use is contraindicated in advanced chronic kidney disease (CKD) [48]. CKD is in turn associated with physiological age-related decline in kidney function and the duration of T2DM [49] (See Supplementary file). Nevertheless, CKD was not associated with B12 deficiency in our data in any of our analyses.

The oldest age group (age ≥ 80 years) —despite being significantly associated with both lower metformin daily dose (described above and below) and lower eGFR (see Supplementary file) —was significantly associated with better T2DM control (lower HbA1c; see Supplementary file). It is thus possible that the lower doses of daily metformin prescribed in older patients were not just due to age-related renal decline, but also to altered pharmacokinetics and tolerability of metformin in the elderly [50]; allowing for less metformin use in the context of better T2DM control.

Unlike these two published studies from Asia [32, 47], we did not find a significant association of age with B12 deficiency at baseline (Table 1). However, after stratifying into 3 age groups and adjusting for the daily metformin dose as a confounder, we found that patients in the very-old group of “≥ 80 years” to be significantly more vulnerable to B12 deficiency when compared to those in the youngest age group of “ < 60 years” (adjusted OR = 2.86; 95% CI, 1.31–6.25, *P* = 0.008). Patients “ ≥ 80 years with T2DM” are precisely most at risk of frailty and even COVID-19 complications as referenced above. That these complications could be further compounded by their increased susceptibility to B12 deficiency makes it more imperative that we case-find and prevent B12 deficiencies in this age group.

After adjustment, we found no significant association of “PPI/H_2_A-use” or “calcium supplementation” with B12 deficiency. PPIs/H_2_As reduce gastric acid digestion and are frequently linked to B12 deficiencies in the literature [5, 6, 16]. However, this lack of consistent association has been reported in the 2 Asian studies referenced above [32, 47] and elsewhere [13, 51]. Vitamin B12 is susceptible to stomach acid degradation and thus needs to form an acid-resistant complex with salivary haptocorrin [7, 25, 52–54]. Haptocorrin subsequently escorts B12 past the acidic stomach to the more hospitable alkaline environment of the duodenum, where it (B12) is transferred to intrinsic factor to form a new complex that is finally taken up by cubulin-amnionless receptors in the terminal ileum for absorption [6]. Herein, PPI/H_2_As could perhaps serve to reduce premature acid degradation of vitamin B12 in the stomach and paradoxically aid in its oral bioavailability instead, hence negating any anticipated net B12-lowering through reduced gastric acid secretion.

“Prescribed B12 supplementation” was present in 188 (32%) of all the patients and was associated with lower odds of B12 deficiencies [55], underscoring its potential utility in the prevention of diabetes-associated B12 deficiencies. Most of the B12 supplementation (112 patients) were in the form of pharmacological preparations typically supplying 200–1000 mcg of B12 per dose (together with vitamins B1 and B6) that were prescribed by hospital specialists or polyclinic physicians. Other preparations included intramuscular cyanocobalamin (1 patient), oral methylcobalamin (5 patients) and over-the-counter multivitamins (70 patients). It is noteworthy that 18.9% of B12-deficient patients had deficiency despite prior prescribed B12 supplementation (Table 1). Although this could be accounted for by non-adherence to prescribed supplements, the national health and nutrition examination survey [56] reported that B12 amounts in the daily recommended doses or general multivitamins are insufficient to correct metformin-associated B12 deficiency. More research is thus needed find an optimal B12 supplementation regimen (and the corresponding adherence of patients to supplementation) [7, 25].

B12 and folate (vitamin B9) complement each other closely, and are both vital components for the proper functioning of “one-carbon metabolism” —a pivotal group of B12-folate-dependent biochemical reactions that are crucial for far-reaching aspects of DNA–protein syntheses, cellular regulation and body repair [4, 57, 58]. Certainly, defects in one-carbon metabolism explain the clinical manifestations seen in B12 or folate deficiencies. As such, our finding of an association between folate deficiency and B12 deficiency implies that the negative clinical impacts of B12 deficiency risk being amplified. The clinical impact could be varied; in a National Health and Nutrition Examination Survey (NHANES) prospective cohort study [59] of 8067 patients with T2DM, both folate and B12 deficiencies were associated with increased cardiovascular disease mortality. Moreover, in a local study [60], higher dietary folate intakes were associated with a lower risk of late-life cognitive impairment (*P*-trend < 0.05).

Almost a quarter (22.3%) of patients were folate-deficient and 8.8% were deficient in both folate and B12. We believe our data to be at least reflective of Singapore where folate fortification of food is not mandatory [55, 61]. A recent local study [37] looked at 577 obese patients with a mean age (± SD) of 40.6 (± 10.3) years and reported a 31% prevalence for folate deficiency (also defined in that study as ≤ 13.4 nmol/L) and 9.5% for B12 deficiency (defined in that study as < 145 pmol/L). Another local study [62] looked at 726 persons from the general population aged 30 to 69 years, and also reported high prevalence of folate deficiency (defined in that study as < 6.8 nmol/L), with the highest prevalence in Indians (men 44.9%; women 36.6%); followed by the Malays (men 45.3%; women 24.5%); and then the Chinese (men 31.4%; women 12.6%). Hence it is vital that B12 deficiency should not be addressed in isolation —but rather in tandem with the possibility of concurrent folate deficiency [36, 63].

We believe that in addition to the absence of folic-acid food fortification, the pervasive hawker (street) food culture in Singapore contributed to the association between folate and B12 deficiencies in our patients. A population-based cross-sectional study of 1170 Chinese women (aged 10–49 years) in Northwest China [64] (an Asian country similarly without mandatory folic-acid food fortification), did also publish an association of folate deficiency with B12 deficiency [64] — attributing this finding to the predominance of simple diets (rich mainly in carbohydrates) in their population.

Unique and central to the diet of Singapore citizens, is the ingrained culture of consuming hawker (street) food. As Singapore industrialized, people needed to eat cheaply and meaningfully because many did not have time to cook [65]; street vendors were regulated and relocated to hygienic facilities close to homes and workplaces in either hawker centres, food courts or coffee shops [65, 66]. In addition to being very affordable [67], eating hawker food has become an opportunity for people from diverse backgrounds to mingle and socialize [67, 68] —earning it (in 2020) an inscription on the UNESCO Representative List of the Intangible Cultural Heritage of Humanity [65, 69].

There are however concerns with the healthfulness [70–74] of hawker foods; many are high in fat, sodium and carbohydrates, while being low in dietary fibre [75–77]. Hawker food preparation—often involving high heat, gravy or soups— could lead to B12-folate thermal degradation, or these water-soluble vitamins’ leaching into the cooking water respectively [78, 79]. Nevertheless, these concerns are often outweighed by hawker foods’ convenience and affordability [71]. This Singapore experience suggests that as Asian cities progress socio-economically, home meal preparation may give way to the convenience of eating out [71].

Data from the National Nutrition Survey 2010 [80] showed that the majority of adult Singapore residents (80.7%) reported eating at hawker centres/food courts/coffee shops (all 3 offer similar dishes) at least twice a week. In addition, nearly half (45.1%) had their meals at hawker centres/food courts/coffee shops six (or more) times a week.

Data from the Singapore Household Expenditure Survey 2012/13 [81] show that the average expenditure on hawker food across income brackets ranged from $291.80 (representing 43% of the total household monthly food expenditure) for the average household in the lowest income quintile, to $408.30 (26% of monthly food expenditure) for those in the highest income quintile. As such, households in the lower income brackets spent a comparatively higher proportion of their monthly food expenditure on hawker food, suggesting a greater reliance on (if not greater relative consumption of) hawker food (See Supplementary Table).

Similarly, monthly household expenditure on “fruits and vegetables” (main dietary sources of folate) was almost double in the highest income bracket ($116.40) compared to the lowest income bracket ($68.30). Despite this, the latter translated to a higher percentage of food expenditure (10%) compared to the richest households (7%) —suggesting a ceiling of affordability of “fruits and vegetables” for the poorer households (See Supplementary Table).

A similar (but larger) disparity is seen in the expenditure on food sources of B12 (Meat, Fish, Seafood, Milk, Cheese and Eggs). While the monthly household expenditure on these foods remained stable (between $185.00 -$196.00) for the top 4 income quintiles, the lowest quintile only spent $136.10, which however translated to 20% (the highest across income quintiles) of food expenditure. This also suggests a limit of affordability of B12-containing foods for the poorest households (See Supplementary Table).

It is notable that 31.6% of resident households in the lowest 20% were headed by persons aged 65 years and over [81]. Although socio-economic status was not collected in our data, 76.9% of our patients were 60 years or older (i.e. within retirement age) and hence were likely to be more prudent with expenditure on daily necessities like food. Nevertheless, we only postulate that our unique local dietary choices (in particular hawker food) could have contributed to our described association between folate and B12 deficiencies, more studies are certainly needed in this regard.

This study is limited by its observational cross-sectional design in that residual confounding cannot be excluded and causality cannot be determined from significant associations. No other biomarker was used to confirm metabolic B12 deficiency, and other potential causes of B12 malabsorption (eg autoimmune or alcohol consumption) were not explored. The relatively small number of patients who were vegetarian could also have conferred a larger confidence interval to the findings for vegetarianism (Table 2). We also based the history of vegetarianism on patient history rather than a validated questionnaire. The histories of supplementation with B12 or calcium were in fact physician-prescribed supplementation and the reasons underlying this common practice were not explored. This could have led to confounding by indication —whereby prescribed B12 supplementation was more likely to be initiated in symptomatic B12-deficient patients much earlier on —thus affecting the true strength of the inverse association of “prescribed B12 supplementation” with B12 deficiency. Nevertheless, the variables used in this study are its strength, for they comprise information that is immediately available and applicable in the primary-care setting.

Although the data was obtained before the introduction of newer drugs for T2DM (eg sodium-glucose transport protein 2 inhibitors and glucagon-like peptide 1 agonists), key aspects of primary-care clinical practice have not changed: metformin (the major determinant of B12 deficiency) remains the mainstay of T2DM treatment [12]; and to lower cholesterol, we are still advocating that patients limit dietary cholesterol to less than 200 mg per day by reducing consumption of eggs [82], shellfish, and organ meats [83–85] (otherwise rich dietary sources of B12) [86]. Moreover, with the projected increase in the prevalence of diabetes and old age in both Singapore and the world at large [2] —coupled with the reduction of food affordability (and consequent narrowing of available food choices) [87, 88] —researching of B12 deficiency in the community has never been more relevant and pressing.

Herein, we hope that the delineated risk factors for B12 deficiencies could serve as a quick guide in a busy clinic to help case-find patients most at risk of deficiencies. In the longer term, we also hope that this manuscript would help guide public healthcare policy —and much-needed future research —with regard to screening and treatment of preventable B12 deficiencies.

## Conclusions

In conclusion, independent of the duration of T2DM, we found a high prevalence of B12 deficiency (27.7%) in our retrospective cohort of primary-care patients with T2DM, with more than a quarter of B12-deficient patients having had repeat screening tests leading to the diagnosis of B12 deficiency. “Metformin daily dose” (an accurate proxy of both cumulative metformin exposure and duration of T2DM); “age ≥ 80 years”; “vegetarianism”; and “folate deficiency” were determinants of B12 deficiency; in contrast, “prescribed B12 supplementation” was associated with a lower odds of B12 deficiency.

We therefore propose that all patients with T2DM in primary care —regardless of the duration of their disease —be screened for B12 deficiency at least once [24] (as we had done in this study). Likewise, we also propose that screening for B12 deficiency be incorporated into the annual routine laboratory review of patients with T2DM—especially when they are prescribed with ≥ 1.5 g/day of metformin; are aged ≥ 80 years old (precisely also the age group most at risk of frailty and COVID-19 complications); vegetarian; and not on prescribed B12 supplementation. Simultaneous evaluation for folate deficiency is essential when addressing T2DM-associated B12 deficiency (Table 3).

**Table 3:** New findings

Clinical Impact
**What is New**
• Risk factors that can help case-find B12 deficiencies (in primary-care patients with T2DM) are identified and compared
• The odds of B12 deficiency increase markedly when “metformin daily dose” is ≥ 1.5 g/day”; and “age is ≥ 80 years”
• Folate (B9) and B12 deficiencies are associated in our cohort; potentially amplifying the complications of deficiencies of these closely related B-vitamins
• “Metformin daily dose” is an accurate proxy of both cumulative metformin exposure and duration of T2DM
**Clinical Implications**
• We now have the framework to case-find and prevent B12 deficiencies in primary-care patients with T2DM

## Supplementary Information

**Additional file 1.**

**Additional file 2.**

## Data Availability

The datasets generated and analysed during the current study are currently not publicly available but are available from the author (Andrew Kien Han Wee) or Department of Research, SingHealth Polyclinics on reasonable request and with permission of the SingHealth Centralised Institutional Review Board.

## Abbreviations

AUROC: Area under the receiver operating characteristic curve
B12: Vitamin B12
BMI: Body mass index
CI: Confidence intervals
CIRB: SingHealth Centralised Institutional Review Board
COVID-19: Coronavirus disease caused by SARS-CoV-2
eGFR: Estimated glomerular filtration rate adjusted for body surface area
FPC: Family Physician Clinic
HbA1c: Glycated haemoglobin
H_2_A: Histamine H2 receptor antagonist
LDL: Low-density lipoprotein
MDRD: Modification of Diet in Renal Disease
OR: Odds ratio
PPI: Proton-pump inhibitor
ROC: Receiver operating characteristic
SD: Standard deviation
T2DM: Type-2 diabetes mellitus
