# Supplementary material for: Determinants of vitamin B12 deficiency in patients with type-2 diabetes mellitus — A primary-care retrospective cohort study
Source: BMC Prim Care. 2023 Apr 20;24:102. doi: 10.1186/s12875-023-02057-x (PMC10116480; doi:10.1186/s12875-023-02057-x)
Supplement: Supplementary file 1 — Additional file 1. [file 12875_2023_2057_MOESM1_ESM.docx]

**Supplementary Figures 1, 2, 3 and 4**

**Fig. 1** Box plot of eGFR by age groups (n=592), showing significantly lower eGFR in the older age groups; (*P* < 0001).

**Fig. 2** Box plot of HbA1c by age groups (n=592), showing significantly lower HbA1c in the older age groups; (*P* < 0001).

**Fig. 3** Box plot of Metformin daily dose (g/day) vs Stages of chronic kidney disease (CKD Stages 1-5) (n=592); (*P* < 001).

**Fig. 4** Box plot of duration of T2DM over the CKD stages (Stage 5 representing the worst kidney function) (n=592); (*P* < 0001).
