# Supplementary material for: Determinants of vitamin B12 deficiency in patients with type-2 diabetes mellitus — A primary-care retrospective cohort study
Source: BMC Prim Care. 2023 Apr 20;24:102. doi: 10.1186/s12875-023-02057-x (PMC10116480; doi:10.1186/s12875-023-02057-x)
Supplement: Supplementary file 2 — Additional file 2. [file 12875_2023_2057_MOESM2_ESM.docx]

**Average Monthly Household Expenditure on HAWKER FOOD Among Resident Households by Type of Goods and Services (Detailed) and Income Quintile (Singapore Household Expenditure Survey 2012/13)**

| Type of Goods and Services | Total  (Singapore Dollars (S$)) | Income Quintile | | | | |
| --- | --- | --- | --- | --- | --- | --- |
|  |  | 1^st^ -20^th^ | 21^st^ – 40^th^ | 41^st^ – 60^th^ | 61^st^ – 80^th^ | 81^st^ – 100^th^ |
| Total Monthly Expenditure on Goods and Services | 4724.5 | 2215.3 | 3530.9 | 4705.1 | 5595.9 | 7575.3 |
| **Total Monthly Expenditure on FOOD (A + B)** | 1188.2 | **677.5** | 1044.8 | 1270.3 | 1374.1 | **1570.9** |
| **(A) Food and Non-alcoholic Beverages (eg tea and coffee)** | 423.8 | 309.6 | 410.6 | 465.5 | 456.1 | 477.4 |
| 1. MEAT | 66.3 | 52.5 | 70.3 | 68.5 | 67 | 73.4 |
| 1. FISH and SEAFOOD | 64.3 | 51.3 | 67.2 | 70.8 | 63.6 | 68.5 |
| 1. MILK, Cheese and Eggs | 49.4 | 32.3 | 48.8 | 57.4 | 54.7 | 53.7 |
| Expenditure on Meat, Fish, Seafood, Milk, Cheese and Eggs  (i + ii + iii) | 180.0 | **136.1** | 186.3 | 196.7 | 185.3 | **195.6** |
| Expenditure on Meat, Fish, Seafood, Milk, Cheese and Eggs as a % of all expenditure on FOOD | 15% | **20%** | 18% | 15% | 13% | **12%** |
| 1. FRUITS | 44.4 | 27.1 | 36.9 | 45.6 | 51.6 | 60.9 |
| 1. VEGETABLES | 51.2 | 41.2 | 50.7 | 54.9 | 53.7 | 55.5 |
| Expenditure on Fruits AND Vegetables (iv + v) | 95.6 | **68.3** | 87.6 | 100.5 | 106.3 | **116.4** |
| Expenditure on Fruits and Vegetables as a % of all expenditure on FOOD | 8% | **10%** | 8% | 8% | 8% | **7%** |
| 1. Oils and Fats | 8.9 | 7.9 | 9.3 | 10.4 | 8.7 | 8.4 |
| 1. Sugar, Jam, Honey, Chocolate and Confectionery | 17.2 | 10 | 14.1 | 18.2 | 20.8 | 22.7 |
| 1. Bread and Cereals | 78.2 | 54.9 | 73.2 | 91.3 | 87.7 | 83.6 |
| **(B) Food-serving SERVICES** | 764.4 | 367.9 | 634.2 | 808.4 | 918 | 1093.5 |
| 1. Restaurants, cafes and pubs | 266.6 | 45 | 121.5 | 234.2 | 322.5 | 609.8 |
| 1. Fast-food restaurants | 45.7 | 24.7 | 44.1 | 54.6 | 56.9 | 48.4 |
| 1. HAWKER food (Hawker Centres, Food courts, Coffee shops, Canteens, Kiosks, Street Vendors.) | 434.9 | 291.8 | 457.5 | 501.6 | 515.2 | 408.3 |
| Expenditure on **HAWKER** **food** as a % of all expenditure on FOOD | **37%** | **43%** | 44% | 39% | 37% | **26%** |
| 1. Other catering services | 9 | 3.3 | 5.9 | 9.1 | 13.3 | 13.5 |
| 1. Food serving services not elsewhere classified | 8.2 | 3.2 | 5.1 | 8.8 | 10.2 | 13.6 |
| Expenditure on **HAWKER food** as a % of all expenditure on FOOD SERVICES | 57% | 79% | 72% | 62% | 56% | 37% |
